# Personal

**Published:** 1903-03

**Authors:** 


### PERSONAL.

DR. FREDERICK PREISS, of Buffalo, who has been ill for several weeks with typhoid fever, is sojourning in Florida during his convalescence, whither he went about the middle of February.

Dr. Marshall Clinton, of 466 Franklin Street, Buffalo, has been appointed attending surgeon at the Buffalo Hospital of the Sisters of Charity.

Dr. Eugene Smith, of Detroit, read a paper before the Buffalo Ophthalmological Club, Thursday evening, February 12, 1903, on Improved methods of cataract extraction.

Dr. DeWitt G. Wilcox, of Buffalo, was re-elected secretary of the Homeopathic Medical Society of the State of New York, during its recent annual meeting at Albany.

Dr. E. G. Danser, of Buffalo, county medical examiner, who has been quite ill recently, has fully recovered and has resumed both his official duties and his professional practice.

Dr. George T. Moseley, of Buffalo, was nominated to the regents as a state medical examiner at the recent annual meeting of the Homeopathic Medical Society of the State of New York.

Dr. Floyd S. Crego, of Buffalo, gave a reception to Dr. Winfield Scott Hall, of the Northwestern University, Chicago, at the Buffalo Club, Wednesday, February 4, 1903, at 4 o'clock. It was numerously attended by physicians and others, and was an enjoyable gathering of representative men.

Hon. St. Clair McKelway, editor of the *Brooklyn Eagle*, has resigned as a regent of the university of the State of New York, on account of ill health, or as he states it: "I take this step with sincere regret after more than twenty years of unsparing service. The reasons moving me concern entirely conditions of health, which admonish me to reduce the demands outside of my profession upon my strength and time. Happily, the board is now so well organised, and so harmonious and effective in its work, that my retirement can adversely affect neither the policy, nor the success, nor the influence of the body as the constitutional trustee of the state for advanced education."

Commenting upon the subject, *The Tribune* of February 18, 1903, says:

The resignation of Mr. St. Clair McKelway, editor of the *Brooklyn Eagle*, from the State Board of Regents will be widely regretted. After twenty years of active, sagacious and loyal service in that capacity, Mr. McKelway is constrained to retire solely because a recent illness, though he has happily recovered, has warned him that he must hereafter devote less time and strength to labors outside of his newspaper office. His colleagues, who urged him to reconsider his decision if he conscientiously could, will greatly miss him, and the public loss is serious. But it is gratifying to know that through his journal he will continue to be a wise counsellor in all matters relating to the schools.

Later—Since the foregoing was reduced to type, Regent McKelway has reconsidered his action and withdrawn his resignation.

In a letter to the Governor, Mr. McKelway stated that his physician had advised him that after a rest he would be able to continue his work, and that having been pressed by many friends not to withdraw from the Board of Regents, he had decided to remain. Governor Odell immediately issued a statement reciting the facts and closing with the following paragraph, with which every person interested in the cause of education will concur:

Mr. McKelway's services have been so valuable to the regents and to the cause of education generally, that I am sure everyone will join in the desire of his colleagues and educators throughout the state that he retain his membership on the board, and I think the state is to be congratulated upon the fact that he will be able to resume his duties as a regent and upon his determination to recall his letter of resignation.

---

Dr. Winfield Scott Hall, of Chicago, dean of the medical department of Northwestern University, was the special speaker at the annual banquet of the Alpha Omega Delta fraternity of the University, held at the Broezel House, Wednesday evening, February 4, 1903. His subject was "Educational Ideals in Medicine."

---

Dr. E. L. Frost, of Buffalo, has been appointed physician to the Erie County Penitentiary by the board of supervisors.

---

Dr. Prescott LeBreton, of Buffalo, has been appointed by the boad of supervisors physician to the Erie County Jail.

---

Dr. Alvah H. Doty, of New York, has been reappointed health officer of that port, and his confirmation by the Senate followed with promptitude. This appointment is for a third term, and speaks well for the efficient management of Dr. Doty during his two previous terms.

